# The Impact of Cell-Intrinsic STAT6 Protein on Donor T Cell-Mediated Graft-Versus-Tumor Effect

**DOI:** 10.3390/ijms26010280

**Published:** 2024-12-31

**Authors:** Xiaoqun Guan, Hope Fury, Priya D. Issuree, Tyler Atagozli, Emory E. McManimon, Peng Shao, Yue Li, Michael Chimenti, Noah S. Butler, Mark H. Kaplan, David E. Elliott, Bruce R. Blazar, M. Nedim Ince

**Affiliations:** 1 Department of Internal Medicine, Division of Gastroenterology and Hepatology, Carver College of Medicine, University of Iowa, Iowa City, IA 52242, USA; xiaoqun-guan@uiowa.edu (X.G.); hope.fury@northwestern.edu (H.F.); tyler-atagozli@uiowa.edu (T.A.); emory-mcmanimon@uiowa.edu (E.E.M.); yueli@som.umaryland.edu (Y.L.); david-elliott@uiowa.edu (D.E.E.); 2 Iowa City Veterans Affairs Medical Center, Iowa City, IA 52246, USA; 3 Department of Internal Medicine, Division of Infectious Diseases, Carver College of Medicine, University of Iowa, Iowa City, IA 52242, USA; priya-issuree@uiowa.edu; 4 Department of Microbiology and Immunology, Carver College of Medicine, University of Iowa, Iowa City, IA 52242, USA; peng-shao@uiowa.edu (P.S.); noah-butler@uiowa.edu (N.S.B.); 5 Iowa Institute of Human Genetics, University of Iowa, Iowa City, IA 52246, USA; michael-chimenti@uiowa.edu; 6 Department of Pediatrics, Herman B. Wells Center for Pediatric Research, School of Medicine, Indiana University, Indianapolis, IN 46202, USA; mkaplan2@iu.edu; 7 Department of Microbiology and Immunology, School of Medicine, Indiana University, Indianapolis, IN 46202, USA; 8 Division of Blood & Marrow Transplant & Cellular Therapy, Department of Pediatrics, University of Minnesota, Minneapolis, MN 55455, USA; blaza001@umn.edu

**Keywords:** STAT6, T helper-2, graft-versus-host disease, graft-versus-tumor effect, bone marrow transplantation, granzyme A, granzyme B

## Abstract

Bone marrow transplantation (BMT) is mainly performed to restore an anti-tumor immune response, called the graft-versus-tumor (GVT) effect, against leukemia, myeloma and lymphoma. This GVT reactivity is driven by donor T cells, and it can also cause lethal graft-versus-host disease (GVHD). We previously demonstrated that the colonization of mice with helminths preserves the GVT response while suppressing GVHD. As the T helper-2 (Th2) pathway is critical to helminthic immune regulation, we asked whether the genetic induction of Th2 signaling in donor T cells can restore helminthic immune regulation after BMT. Our studies utilized transgenic donor T lymphocytes that overexpress a constitutively active form of the Th2-associated transcription factor STAT6. Constitutively active STAT6 sustained the GVT response without causing severe acute GVHD, where transgenic T cells generated robust quantities of cytotoxic proteins important in GVT response, such as granzymes A and B, interferon-γ and Fas ligand, in addition to generating high quantities of Th2/regulatory cytokines. Bioinformatic analysis based on chromosome immune precipitation experiments indicated that STAT6 stimulates the expression of granzymes directly. Thus, in preserving the GVT response without causing GVHD mortality, our results indicate the therapeutic potential of restoring helminthic immune modulation by targeting STAT6 and STAT6-dependent T cell maturation.

## 1. Introduction

Bone marrow transplantation (BMT) is mainly performed to cure a hematological malignancy, such as leukemia, myeloma or lymphoma [1]. In addition to bone marrow or hematopoietic stem cells, donor cell inoculum also includes peripheral T cells that restore the graft-versus-tumor (GVT) effect and thereby prevent tumor recurrence after transplantation [2,3]. The drawback of this approach is the simultaneous attack of host tissues by donor T cells, which can result in lethal and devastating graft-versus-host disease (GVHD). Various approaches that carry on donor T cell transfer with hematopoietic stem cells are under investigation to sustain the GVT effect without causing severe and lethal GVHD [1].

Exploratory strategies that suppress GVHD while preserving the GVT effect comprise the induction of the T helper-2 (Th2) pathway [4]; the latter includes the cytokine interleukin-4 (IL4), proteins of IL4 receptor (IL4R) complex such as IL4Rα chain or the transcription factor STAT6. IL4 stimulates the Th2 signaling by activating STAT6, and the IL4-dependent regulation of acute GVHD requires donor T cell STAT6 signaling in some models [5,6,7]. The donor T cell inoculum also contains Foxp3+ CD4 regulatory T lymphocytes that exert suppressive effects on GVHD while preserving the GVT effect [8]. In this context, the IL4- and Th2-dependent regulation of GVHD promotes the expansion of donor Foxp3+ Tregs and the preservation of the GVT response [5,6,9,10,11].

In an in vivo milieu of regulated GVHD, the direct impact of donor Th2 polarization on GVT response is less well characterized and requires further investigation despite the presence of preliminary evidence for a possible beneficial effect [12,13]. Th2 reactivity or IL4 can promote anti-tumor response in some models, whereas donor T cell Th2 reactivity can have an adverse effect on anti-tumor immunity in others. When intestinal colonization with helminths was used to stimulate the Th2 pathway and regulate GVHD in our laboratory, the GVT response was preserved [9]. In this helminth-induced model, we found that GVHD regulation is Th2-dependent, associated with the induction of donor T cell IL4 secretion (thus Th2 maturation) and with the maintenance of GVT response.

To determine whether the GVT response can be preserved without causing GVHD-related mortality by directly augmenting Th2-dependent responses, we made use of a mouse model whose T lymphocytes express a constitutively active form of STAT6, in which the expression of the transgene is driven by human CD2 locus control region [14]; this model has historically permitted efficient in vivo protein expression in mice [15]. In this mouse, the alanine substitution of two neighboring amino acids in the STAT6 coding sequence (valine (V547) and threonine (T548)) gives rise to T cells that have a constitutively phosphorylated and active STAT6 protein (designated as STAT6VT) [16]. Here, we show that the STAT6VT transgene triggers the generation of high quantities of Th2 (IL4 and IL10) cytokines in T cells, preserves a regulated Th1 cytokine interferon-γ (IFNγ) secretion and produces cytotoxic effector molecules associated with potent GVT response. Strikingly, the adoptive transfer of STAT6VT+ T cells with BMT preserves the GVT response without causing severe GVHD or GVHD-related mortality.

## 2. Results

### 2.1. The Impact of STAT6VT+ Donor T Cells on Alloreactivity and Cytokine Production After BMT

To determine whether helminth colonization per se (from our previous studies) or the associated Th2-dependent effects were responsible for the observed separation of GVHD and GVT, we used transgenic donor T cells overexpressing a constitutively active STAT6 protein (STAT6VT) [14]. The T cell-specific transgenic STAT6VT mouse was successfully used to establish disease models and to investigate atopic dermatitis-like skin disease, allergic pulmonary inflammation with eosinophilic infiltration [17] or Tregs [18,19].

**Figure 1:**
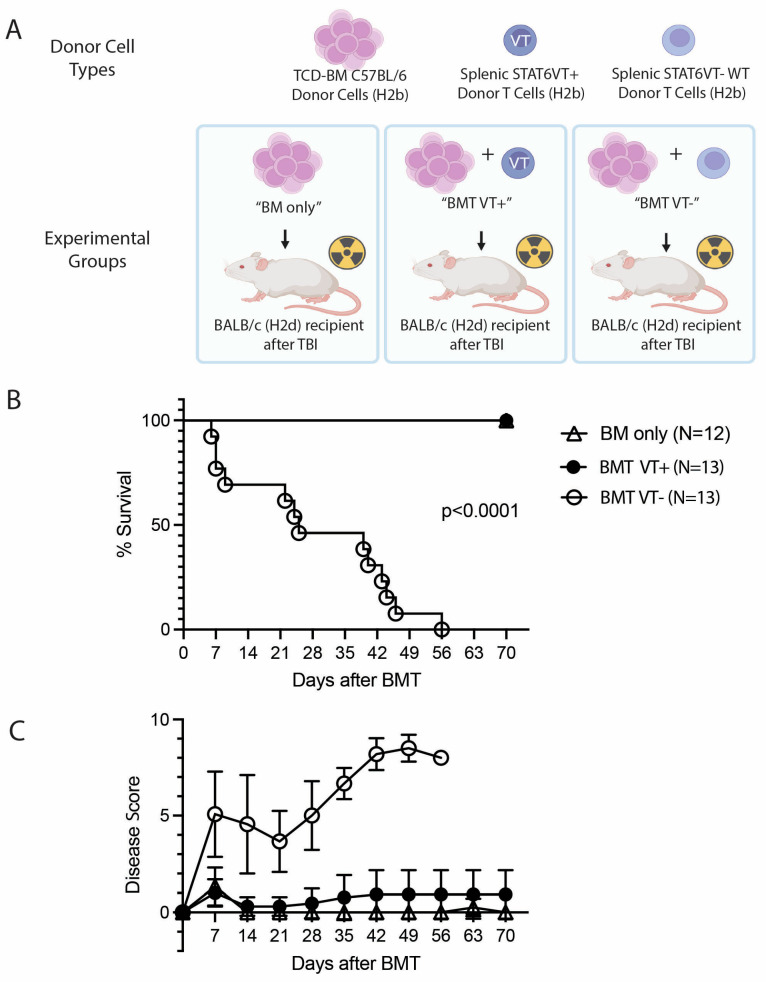

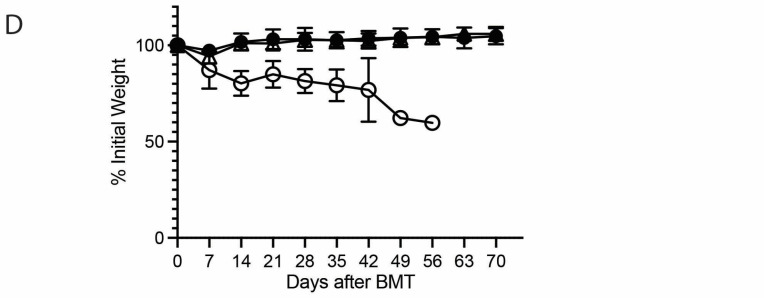
Transfer of STAT6VT donor T cells does not cause GVHD-related mortality. (**A**) Experimental design with different donor cell types administered to BALB/c after total body irradiation (TBI) and the MHC haplotypes of donor and the recipient (TCD-BM: T cell-depleted bone marrow). (**B**) Kaplan–Meier survival curves (**C**) acute GVHD disease scores and (**D**) weight change (% change compared to original weight of survivors) of WT BALB/c BMT recipient mice that received WT C57BL/6 TCD-BM cells only (BM only group; triangles; N (total number of mice) = 12), WT C57BL/6 TCD-BM cells with STAT6VT+ donor T cells (BMT VT+ group; filled circles; N = 13) or WT C57BL/6 TCD-BM cells with STAT6VT− (WT) donor T cells (BMT VT− group; open circles; N = 13). Surviving mice were observed for 70 days after BMT. Data are pooled from 3 independent experiments with “N” representing the total number of mice in each group. Statistical analyses were performed using log-rank (Mantel–Cox) test in survival curves and Student’s unpaired *t*-test for multiple comparisons of disease score and weight change except for time points in which a single data point is evident. Statistical difference in survival between the BMT VT− group and other groups was significant and shown on graph. Statistical differences between disease scores and weight changes between groups are displayed in Supplementary Materials.

In a BMT and MHC I/II major mismatch (H2b→H2d) GVHD model after myeloablative preparation, BALB/c recipients (H2d) received T cell-depleted bone marrow cells from C57BL/6 (H2b) and splenic donor T cells from STAT6VT+ (H2b) mice (Figure 1A). All mice, which received STAT6VT-negative (STAT6VT−; WT; BMT VT− group) donor T cells (lethal GVHD control) displayed signs of severe acute GVHD and died (Figure 1B,D). By contrast, all BALB/c recipients of STAT6VT+ donor T cells (BMT VT+ group) survived the 70-day observation period, and the GVHD disease score in these recipient mice remained minimal. Similar to the latter group, BALB/c mice that only received TCD-BM without splenic T cells (no GVHD control; BM-only group) also survived without showing signs of GVHD (Figure 1B,D). Weight loss was also evident in BMT VT− and not in other groups (Figure 1D). These results revealed that administration of splenic STAT6VT+ donor T cells does not cause acute lethal GVHD.

**Figure 2:**
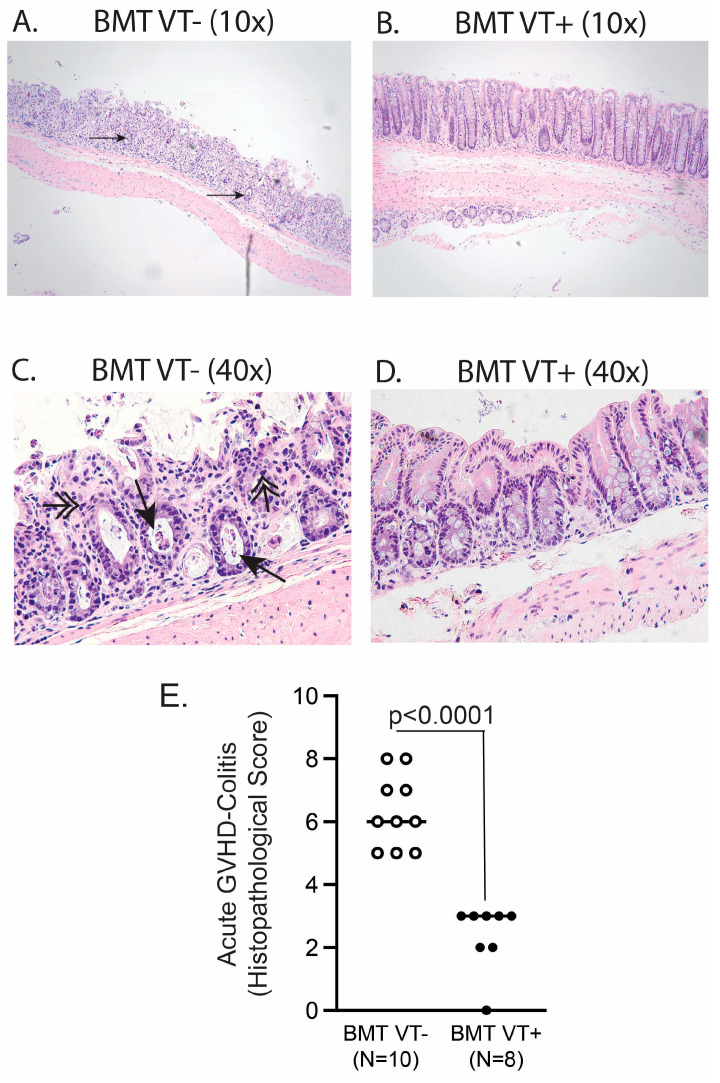
Transfer of STAT6VT+ donor T cells causes mild GVHD-associated colitis. Representative histopathological analysis of the colon isolated 6 days after BMT (10× of original magnification (**A**,**B**) and 40× of original magnification (**C**,**D**)) from BMT VT− (**A**,**C**; N = 10) or BMT VT+ (**B**,**D**; N = 8) groups. Black arrows represent apoptotic crypt abscesses, whereas double-head arrows indicate apoptotic bodies. (**E**) Cumulative data from two independent experiments with disease score of a single mouse represented as a single dot and the mean as a bar. The total number of samples in each group is shown as “N”. Differences between groups were determined using *t*-test and displayed.

Parallel groups of BALB/c mice then received the same treatment as before (Figure 1), and recipients were sacrificed 6 days after BMT for histological quantification of GVHD-associated colitis (Figure 2). As observed by low-power field magnification (Figure 2A,B) and high-power magnification (Figure 2C,D), the administration of STAT6VT-negative (STAT6VT−; WT) T cells was associated with severe GVHD-related colitis (Figure 2A,C). Such changes were characterized by apoptotic crypt abscesses (black arrow) and apoptotic bodies (double-head arrow). In contrast, the administration of STAT6VT+ donor T cells was not associated with severe colitis (Figure 2B,D), which was also reflected in a combined histopathological score (Figure 2E). Cumulatively, these results were consistent with survival, weight curves and GVHD disease scores (Figure 1) and confirmed the observations that STAT6VT+ donor T cell administration is not associated with severe GVHD-associated major end-organ damage.

**Figure 3:**
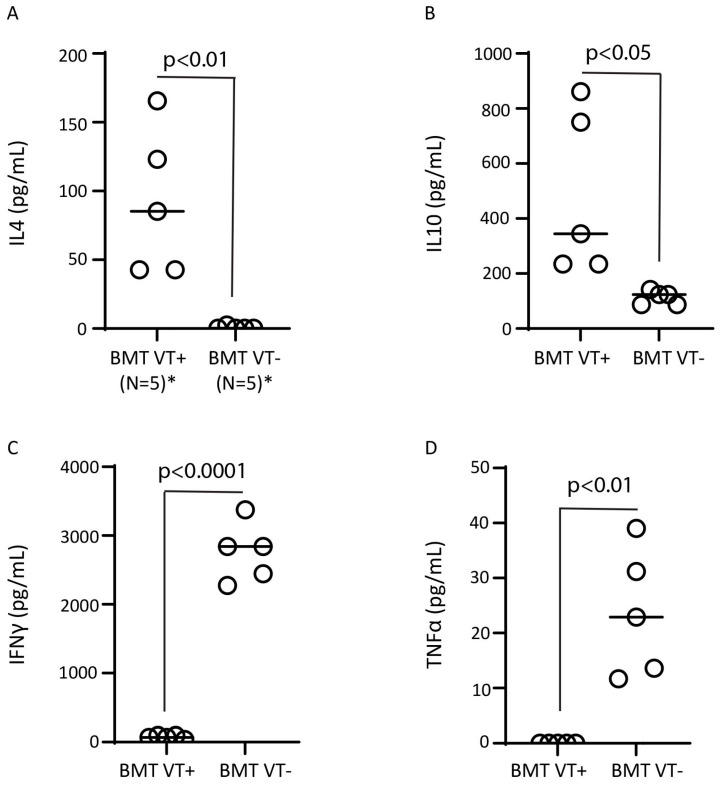
Transfer of STAT6VT+ donor T cells promotes regulatory and Th2 cytokine generation and suppresses inflammatory cytokines. Serum was purified from mice 6 days after BMT and analyzed for IL4 (**A**), IL10 (**B**), IFNγ (**C**) and TNFα (**D**) cytokine content by ELISA. Cumulative data from two independent experiments with each dot representing the mean cytokine content from a single mouse; “N” is the total number of samples in each group; (*) N is 5 for each dot column graph on each panel; *p*-value between BMT VT+ and BMT VT− groups is displayed on each panel.

The STAT6-driven Th2 pathway and immune regulatory cytokines (IL10, TGFβ) are associated with the regulation of Th1 cytokine IFNγ production and the mitigation of acute GVHD, the severity of which is more dominantly driven by Th1 cytokines [4]. When serum from BMT recipient mice (Figure 3) was analyzed for concentrations of various cytokines, we observed a dramatic increase in Th2 cytokine IL4 and regulatory/Th2 cytokine IL10 concentrations in the serum of the BMT VT+ group, while the Th1 cytokine IFNγ was significantly downregulated (Figure 3A−C). As IFNγ originates from T cells (after BMT, mostly from donor T cells in our model), these results suggest that the transfer of STAT6VT+ donor T lymphocytes is associated with regulated donor T cell inflammatory cytokine IFNγ generation. Regulated IFNγ generation is associated with highly regulated acute GVHD based on data from GVHD disease and histopathological GVHD-associated colitis scores (Figure 1 and Figure 2). Similarly, the proinflammatory cytokine TNFα remained low in recipients of STAT6VT+ donor T cells compared to recipients of STAT6VT− donor T cells (Figure 3D).

### 2.2. Donor Treg Frequencies After STAT6VT+ T Cell Addback and BMT

**Figure 4:**
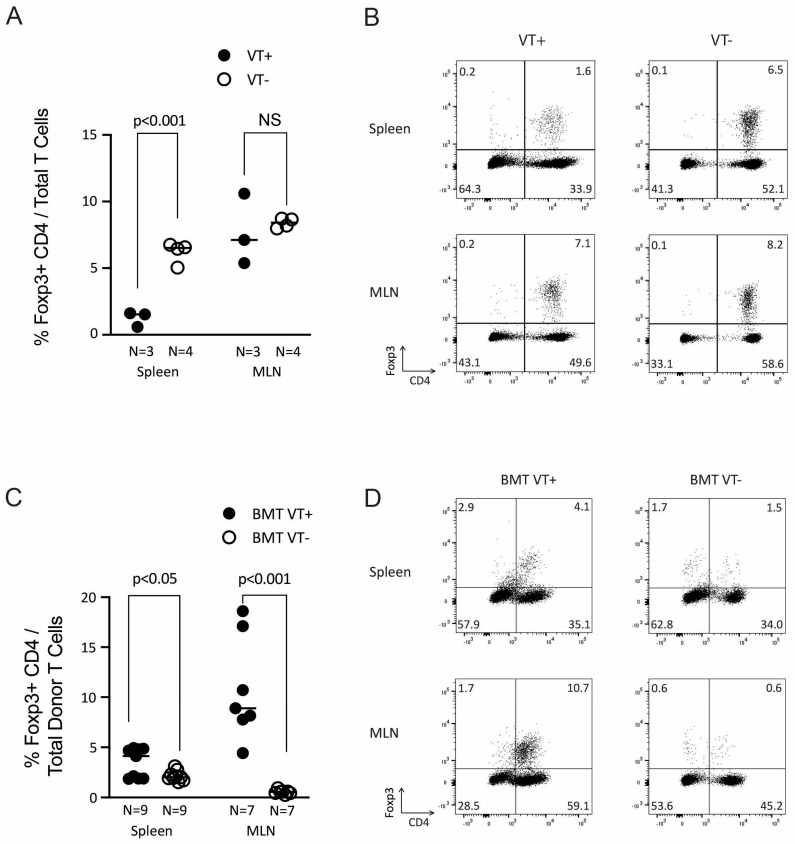
Addback of STAT6VT+ T cells is associated with expansion of donor Foxp3+ CD4 Tregs in BMT recipients. (**A**) Percentage of Foxp3+ splenic and MLN CD4 Tregs from STAT6VT+ (VT+) mice (N = 3) compared to their STAT6VT− (VT−; N = 4) counterparts. Each dot represents percentage from a single mouse; “N” is the total number of mice, and the *p*-value is as displayed on the graph. (**B**) Representative dot plot of CD4 vs. Foxp3 analysis for samples from panel A. Cells are gated on CD3 positive lymphocytes. Percentage of events in each quadrant is displayed. (**C**) Percentage of donor Foxp3+ CD4 Tregs among total donor T cells in spleen and MLN of BMT VT+ and BMT VT− mice. Mice were sacrificed 6 days after BMT, spleen and MLN cells were analyzed for H2b, CD3, CD4 and Foxp3 staining by flow cytometry. Cumulative data from multiple experiments where each dot represents a single mouse in spleen samples and where each dot represents pooled samples from two to three mice in MLN samples; “N” is the total number of dots (independent samples) in each group (BMT VT+ vs. BMT VT−); N = 9 for each group of independent spleen and N = 7 for each group of independent MLN samples; *p*-value as displayed on graphs. (**D**) Representative dot plot of CD4 vs. Foxp3 analysis for samples from panel C. Cells are gated on CD3 and H2b (donor) positive lymphocytes. Percentage of events in each quadrant is displayed.

Our previous studies indicated that after helminth infection, the expansion (or possibly new differentiation) of GVHD-regulating donor Tregs is dependent on TGFβ, which in turn is induced by the Th2 pathway [6,9,10,11]. Therefore, we investigated whether STAT6VT transgene expression promotes the expansion of donor (STAT6VT+) CD4 Tregs after BMT. We first analyzed the frequency of Foxp3+ CD4 Tregs among all splenic T cells (in other words, the donor T cell population) before transfer and found that the Treg percentage in STAT6VT+ mice is lower compared to STAT6VT− WT mice (Figure 4A,B).

To determine whether Tregs from STAT6VT+ mice are increased in BMT recipients undergoing a GVHD reaction, we administered splenic donor T cells from STAT6VT+ mice or their STAT6VT− WT counterparts along with TCD-BM cells from C57BL/6 WT mice into BALB/c recipients after TBI. Six days after BMT, we observed a modest but statistically significant increase in the percentage of Foxp3+ donor CD4 Tregs among total donor T cells in the spleen and a robust and dramatic expansion of donor CD4 Tregs in MLN of BMT VT+ recipients (Figure 4C,D). These results suggest that active STAT6 signaling can play a role in either the expansion of preformed CD4 Tregs or their differentiation from T lymphocyte populations during GVHD. Based on the difference between donor Treg frequency in the spleen versus MLN, the Treg expansion/differentiation can depend on other gut-derived immune modulatory factors. An analogous higher MLN Treg percentage in STAT6VT+ mice (Figure 4A,B) further confirms that gut-derived factors can promote the expansion or differentiation of Tregs, in synergy with STAT6 signaling. In addition to BMT models after helminth infection, our results are also consistent with other observations indicating the importance of the Th2 pathway in the expansion of GVHD-regulating donor Tregs [5].

### 2.3. The Impact of STAT6VT+ Donor T Cells on Graft-Versus-Tumor (GVT) Response

**Figure 5:**
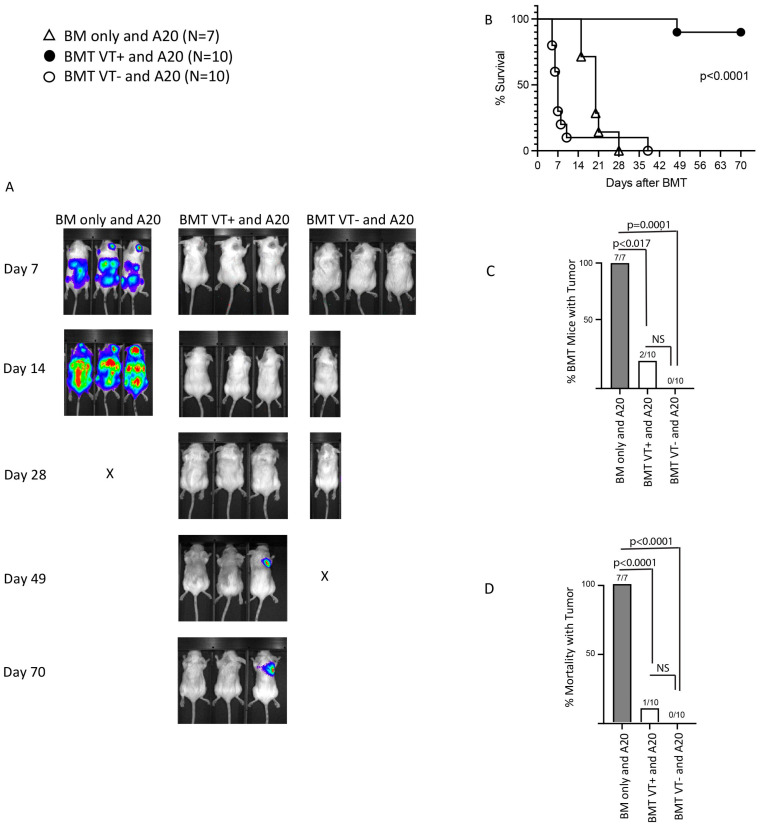
Addback of STAT6VT+ T cells preserve the GVT effect. (**A**) Representative example of bioluminescence imaging of BMT mice over the time course of a single experiment. Data are acquired at designated time points (left) after BMT and the administration of A20-luc leukemia cells. (**B**) Kaplan–Meier survival curve of mice from three different groups, (1) WT BALB/c recipients of TCD-BM donor cells from WT C57BL/6 mice and A20 cells (BM only and A20; triangles; N = 7), (2) WT BALB/c recipients of TCD-BM donor cells from WT C57BL/6 mice, splenic donor T cells from STAT6VT+ mice and A20 cells (BMT VT+ and A20; black circles; N = 10), (3) WT BALB/c recipients of TCD-BM donor cells from WT C57BL/6 mice, splenic donor T cells from STAT6VT− mice and A20 cells (BMT VT− and A20; white circles; N = 10); “N” is the cumulative number of mice in each group (cumulative data from 3 independent experiments). Statistical analysis of survival was performed using log-rank (Mantel–Cox) test; *p*-value is as displayed between BMT VT+ and other groups. (**C**) Column graph displaying percentage of mice that developed tumors in each group, as detected by bioluminescence during the course of the experiment. Number of mice that developed tumors over the number of total mice for the group is displayed on the top of each column. (**D**) Column graph displaying percentage of mice that died with tumor in each group as detected by bioluminescence and mortality during the course of the experiment. Number of mice that died with a tumor over the number of total mice for the group displayed on the top of each column. Fisher’s exact test was used as the statistical method (**C**,**D**); Bonferroni correction was applied to determine the difference between the three groups (adjusted *p*-value = 0.05/3 = 0.017); *p*-value between panels as displayed; NS: not significant.

Donor T cells can recognize alloantigens on host tissues that culminate in GVHD and on host tumor cells that provide a GVT effect, which may spare the recipient from succumbing to the underlying malignancy, as first described by Mathe et al. [20]. Therefore, we asked whether STAT6VT+ T cells sustain a potent GVT response utilizing the A20 lymphoma cell line (H2d) of BALB/c donor origin (syngeneic with the recipient) in our GVHD model. BALB/c recipient mice of TCD-BM donors (“BM only” group) rapidly succumbed to extensive tumor growth, and BALB/c recipients of donor T cells from WT mice (BMT VT− mice) died of severe acute GVHD without the development of tumors detectable by bioluminescence (Figure 5A,B). Notably, BALB/c recipients of STAT6VT+ donor T cells (BMT VT+ group) did not develop acute lethal GVHD and still showed a potent GVT effect over the 70 days of experimentation (Figure 5A,B), attesting to a potent STAT6VT+ T cell-mediated GVT response. Interestingly, 2 of 10 BMT VT+ mice showed tumor development. Although the tumor was progressive in one recipient and associated with the death of the BMT recipient, the other recipient showed a persistent/slow-growing tumor and no mortality (Figure 5C,D). Taken together, our results show that overexpression of a constitutively active STAT6 gene in donor T cells abrogates GVHD while retaining a potent GVT effect and acts in the same direction with helminths, regulating GVHD and preserving the GVT.

### 2.4. The Role of Constitutively Active STAT6 in the Generation of Cytokines and the Expression of Antitumor Effector Genes

**Figure 6:**
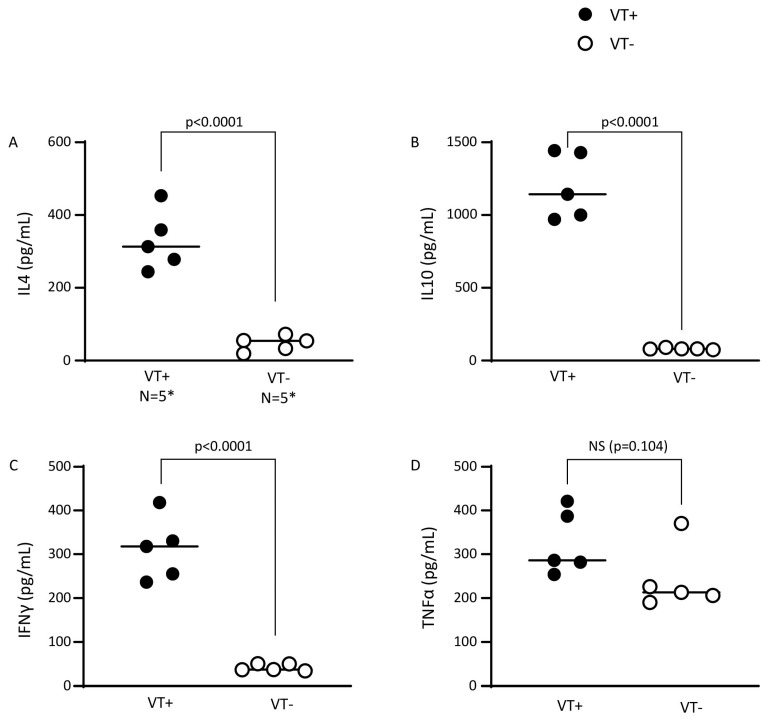
STAT6VT+ T cells generate regulatory and inflammatory cytokines. Purified splenic T cells from STAT6VT+ (VT+) and STAT6VT− (VT−) mice were cultured in vitro and culture supernatants were harvested 48 h late for ELISA (IL4 (**A**), IL10 (**B**), IFNγ (**C**), TNFα (**D**)). Cumulative data from two independent experiments where each dot represents mean cytokine content from in vitro-stimulated samples of splenic T cells from a single mouse; “N” is the total number of mice in each group; * N = 5 for each dot plot column; *p*-value between VT+ (N = 5) vs. VT− (N = 5) T cells as displayed on each panel; NS: nonsignificant.

To obtain more insight into how a constitutively active STAT6 protein in donor T cells drives the GVT response without causing GVHD, we analyzed the cytokine production pattern of splenic donor T cells from STAT6VT+ mice and their WT counterparts. As compared to splenic T cells from STAT6VT− WT (control) mice, splenic STAT6VT+ T cells produced high quantities of Th2 cytokine IL4 and regulatory/Th2 cytokine IL10 after in vitro stimulation with anti-CD3/28 (Figure 6), consistent with our previous results that demonstrated the STAT6-driven generation of immune regulatory TGFβ [10] and the TGFβ-dependent generation of IL10 [21]. Interestingly, STAT6VT+ T cells produced significant quantities of IFNγ, suggesting that the expression of a constitutively active STAT6 protein can activate them [14]. On the other hand, in vitro-stimulated (anti-CD3/28) TNFα production in splenic T cell cultures from STAT6 VT+ mice was comparable to T cell cultures from their WT counterparts and remained at baseline. These findings suggest that different mechanisms promote or suppress IFNγ and TNFα production.

**Figure 7:**
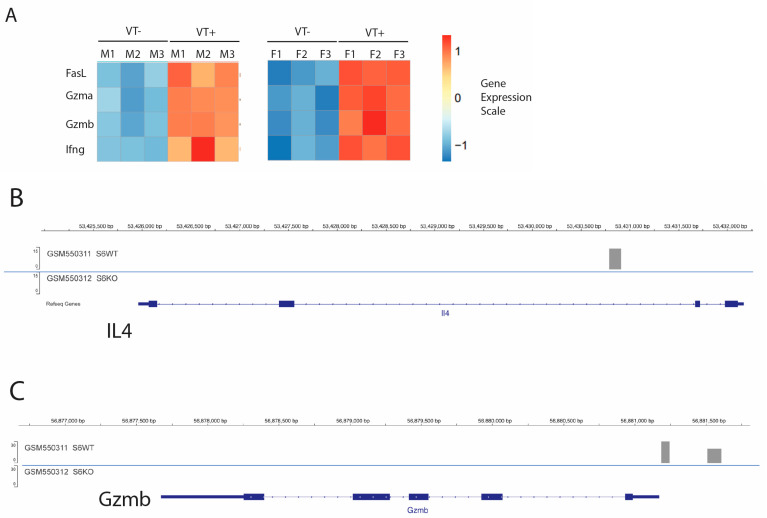

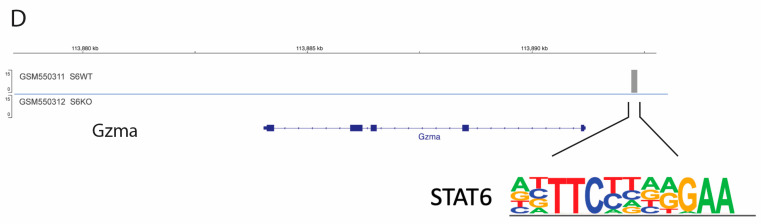
STAT6 directly binds and induces the expression of anti-tumor effector T cell proteins. (**A**) The expression pattern of anti-tumor effector proteins (Granzyme A, granzyme B, FasL and IFNγ) of splenic T cells from STAT6VT (VT+) mice compared to their WT (VT−) counterparts from male (M) and female (F) mice. Each column (M1–M3; F1–F3 for VT+ or VT− samples) represents an independent sample derived from an individual RNAseq analysis of splenic T cells from a single mouse. Expression scale is also shown. (**B**–**D**) Computational analysis of STAT6 binding on genes, IL4 (**B**), GzmB (**C**) and GzmA (**D**) based on data from previous chromosome immune precipitation experiments. The STAT6 binding motif was also shown attached to (**D**).

To identify genes potentially associated with the GVT response, we performed bulk RNAseq experiments from FACS-sorted splenic T cells of STAT6VT+ mice (N = 6) and their STAT6VT− WT (N = 6) counterparts (Figure 7). To determine whether these expression changes correlated with STAT6 binding at these loci, we mined publicly available STAT6 ChIP-Seq datasets previously performed in Th2-polarized murine T cells (GSE22104) [22,23] (Figure 7). Strikingly, STAT6 ChIP-Seq peaks were prominent at the regulatory elements of granzyme A and granzyme B gene loci in WT cells but absent in the STAT6-/- counterparts. STAT6 was also bound to the IL4 gene locus, as previously reported (Figure 7) [23,24]. Together these results suggest that granzyme A and B expression are likely driven by means of in cis transcriptional regulation by STAT6. Granzyme A and granzyme B are elements of the perforin (granule exocytosis) pathway and in the context of the importance of the perforin pathway and FasL, which likely coordinate to execute the GVT effect [25], our results link STAT6-mediated gene transcription directly to cytolytic molecules involved in conferring anti-tumor immunity.

## 3. Discussion

The antitumor effects of allogeneic donor hematopoietic cells were first reported more than 50 years ago [21,26], with different immune cells contributing to them and donor T cells being the major players [27]. Unfortunately, the beneficial GVT response is associated with the in vivo activation of donor T lymphocytes after transplantation, and this increases the risk of lethal and devastating GVHD. In this framework, although recent studies have demonstrated that certain posttransplant immune suppression strategies (e.g., cyclophosphamide) alleviate GVHD while preserving the GVT effect [3,28], balancing GVHD and GVT remain pertinent concerns in clinical practice. Complex mechanisms are believed to drive the antitumor immunity by donor T cells, thereby separating the GVT effect from GVHD. The present study focuses on Th2 response to address this complex and divided T cell reactivity.

Here, we present evidence that a constitutively active STAT6, which is a Th2-associated transcription factor, in donor T cells drives a potent GVT response without causing severe GVHD. In this context, the constitutively active STAT6 drives the expression of granzyme A, granzyme B, FasL and IFNγ proteins, which can be critical to donor T cell-mediated anti-tumor (GVT) immunity [29]. Moreover, our results indicate that STAT6 can promote granzyme A and granzyme B expression by directly binding to their regulatory sequences. Last, we also showed that the constitutive STAT6 activity in donor T cells has a positive impact on the expansion of donor Foxp3+ Tregs after BMT, which protects from GVHD without eliminating the GVT effect [30].

Previously, we also observed protection from GVHD without elimination of GVT after helminth infection [9]. Based on our previous studies, the helminthic regulation of GVHD is dependent on Th2 cytokine IL4 generation by recipient cells, which in our further studies in IL4Rα-/-, T cell-specific IL4Rα-/- and STAT6-/- BMT recipients [6,10,11] appears to act on other BMT recipient cells (more specifically on recipient cell IL4 receptor and STAT6) to condition the host and regulate GVHD. As we also showed before, helminths induce the Th2 pathway and IL4 production by donor T cells [9]. In this current manuscript, we focused on the donor T cell Th2 pathway: First, we showed that constitutive STAT6 activity in T donor cells promotes Th2 maturation with the robust expression of IL4, IL10 and, as we showed previously, the immune regulatory cytokine TGFβ [10]. Next, we demonstrated that the induction of the donor T cell Th2 pathway has a potent impact on the GVT response. Our results advance our previous observations in that the overexpression of a constitutively active STAT6 protein in donor T lymphocytes is sufficient to mimic the helminth effect by separating GVHD suppression and the GVT effect [9].

The separation of GVHD and GVT by donor Th2 cells was tested in the past, where studies have addressed the role of Th2 proteins upstream of STAT6, such as IL4. The adoptive transfer of donor T cells after in vitro propagation with IL4 stimulation showed the short-term GVT effect by donor IL4- (Th2)-conditioned CD8 T cells [12,31]. IL4 activates STAT6, and STAT6 is believed to exert its effects by two mechanisms: First, STAT6 binds to promoter sequences and stimulates transcription. Second, STAT6 coordinates the chromatin accessibility of IL4-responsive genes [32]. In this current work, demonstrating the STAT6-mediated, long-term GVT effect, we investigated whether the same STAT6-dependent pathways promote the expression of proteins associated with anti-tumor immunity, where the mechanistic details of the potent GVT effect by STAT6VT transgenic T cells came from RNAseq experiments and data analysis based on previous observations using chromosome immune precipitation [23]. In addition to inducing the expression of FasL by an unknown mechanism, our evidence suggests that STAT6 binds to and activates the transcription of granzyme A and granzyme B, which are elements of the perforin (granule exocytosis) pathway in inducing target cell apoptosis/killing.

This information can be critical to understanding the Th2-mediated separation of GVT from GVHD, as it is also observed in models of NKT cell-mediated GVHD regulation [5,33] or after helminth infection [9], and it deserves further attention because donor T cells make differential use of cytolytic pathways in exerting anti-host (GVHD) vs. anti-tumor (GVT) cytotoxicity [34]. Furthermore, based on results from previous BMT models, perforin and FasL [33] appear to act together to preserve the GVT without causing GVHD [25,35]. Such a STAT6-mediated preservation of GVT response without causing severe, lethal acute GVHD also contributes to our understanding of how the conditioning of the donor T cells with IL18 can preserve GVT response without causing GVHD because previous studies have shown that IL18 conditioning regulates GVHD in a STAT6-dependent manner [36], and the same conditioning proceeds via the perforin pathway to preserve GVT immunity [37]. Another candidate gene in this context is Nfil3, which is activated by STAT6 and promotes Th2 maturation [38]. Moreover, Nfil contributes to target cell killing by cytotoxic T cells [39] and its role in GVT response deserves further investigation. The Th2 (STAT6)-dependent separation of GVHD from the GVT effect may be a direct impact of STAT6 on gene transcription (such as granzymes) or the result of the STAT6-dependent induction of signaling pathways associated with T cell development or Th2 polarization. The use of STAT6VT-transduced wild-type peripheral T cells as donor T lymphocytes or the inducible and selective deletion of STAT6 in the same cell group can differentiate between these possibilities and advance our understanding of STAT6-mediated separation of GVHD from GVT in future studies.

A potent GVT response while regulating GVHD is also exerted by Foxp3+ CD4 regulatory T cells (Treg) [30]. We demonstrated here that the overexpression of a constitutively active STAT6 protein in donor T cells has a robust effect on the expansion of donor Foxp3+ CD4 Tregs in vivo in BMT recipient mice. This result is consistent with our previous observations demonstrating the dependence of expansion of GVHD-regulating donor Foxp3+ CD4 Tregs on induced Th2 pathway, more specifically on the recipient’s Th2 pathway in previous studies [6,10,11], which is associated with donor T cell Th2 cytokine generation [9]. Constitutively active STAT6-dependent expansion was significantly more pronounced in MLN than in the spleen, suggesting that gut-derived immune modulatory signals, even in the absence of helminth colonization, can still expand donor Tregs in a STAT6 (Th2)-dependent manner and attest to a possible critical role of gut immunity in altering the course of GVHD. STAT6 was proposed to provide a nonredundant signal to Treg expansion [19], which we showed to depend on STAT6- (Th2)-dependent TGFβ activation after helminth infection [6,9,10,11]. According to a recent transcriptomic analysis of donor T cells co-transplanted with donor Tregs in a GVHD model, the addback of Tregs augmented the expression of some Th2 signature genes in donor T cells [40]. The STAT6 dependency of the Treg-mediated separation of GVHD and GVT and the induction of expression of anti-tumor proteins remains to be established.

In this context, the cell-specific expression of granzymes in Foxp3+ Tregs or other T cells deserves further investigation. With cumulating data attesting to a critical role of granzymes in Treg-mediated immune modulation, such a direct genetic approach will advance our understanding of the role of STAT6-driven granzyme expression in donor T lymphocyte subsets. Performing these proposed experiments in other tumor models, for example, additional cell lines syngeneic with the recipient beside A20, or in BMT recipients after retroviral transduction of recipient bone marrow cells with oncogenes, will determine how much we can generalize our observations.

In a delicate balance between immune regulation and achieving robust anti-tumor immunity after BMT, it is possible that STAT6-dependent gene expression can lead to diverse outcomes in different cells. Such a possibility is supported by the distinct outcomes of STAT6-dependent gene expression after Th1 vs. Th2 maturation [23] or in Th17 cells switching to Th1 or Th2 and co-expressing different cytokines according to their microenvironment [41]. In the complex in vivo ecosystem after BMT, in which several other variables have an effect on the outcome (for example, gut microbiome composition influencing the predisposition to GVHD [42], vitamin D promoting Th2 maturation in a STAT6-dependent manner [43] and coordinating immune reactivity with the microbiome [44]), further characterization of the function of STAT6 in T cell immunity merits further exploration. These efforts will advance our knowledge of mechanisms of helminthic immune regulation and helminth-induced separation of GVHD and preservation of a long-term GVT effect. Finally, in the era of genetic manipulation of T cells in clinical BMT practice (e.g., CAR-T therapy), understanding the impact of STAT6 expression on T cell function is expected to potentiate data obtained from anti-helminthic host response for translational medicine [45,46,47,48] and be utilized in various treatment options, like gene therapy.

## 4. Materials and Methods

### 4.1. Mice

Wild-type (WT) C57BL/6 (MHC haplotype: H2b) and WT BALB/c (H2d) mice were received from Jackson Laboratory (Bar Harbor, ME, USA) and maintained at the pathogen-free facilities of the University of Iowa. STAT6VT mouse (H2b) was described previously [10,14]. All mice were maintained and used in accordance with the guidelines of the University of Iowa Animal Care and Use Committee.

### 4.2. Donor Cell Purification for GVHD Induction

Bone marrow (BM) cells were obtained from the tibias and femurs of uninfected, 5–8-week-old WT C57BL/6 mice. Samples were depleted of T cells (T cell-depleted; TCD) using mouse pan T cell beads (Dynabeads Mouse Pan T (Thy 1.2), Invitrogen), as described previously [11]. Splenic T cells were prepared from splenic mononuclear cell suspensions of uninfected, 5–8-week-old C57BL/6 mice using T cell enrichment with T lymphocyte (CD3+) isolation kit (Dynabeads Untouched Mouse T cells, Invitrogen).

### 4.3. Total Body Irradiation (TBI), GVHD Induction and Quantitation of Disease Score

Our studies utilized an acute lethal GVHD model with MHC class I and class II (MHC I/II; H2b→H2d) mismatch. WT BALB/c recipients (H2d) were subjected to TBI using a Cs^137^ source (total of 850 cGy given in two doses and four hours apart). 10 × 10^6^ T cell-depleted bone marrow (TCD-BM) cells from C57BL/6 donors (H2b) with or without 1.5 × 10^6^ purified splenic T lymphocytes from C57BL/6 WT or STAT6VT donors (both H2b) were administered intravenously via retroorbital injection. Disease severity was scored based on animal weight, posture, activity, fur texture and skin integrity [49,50,51]. Each element of these criteria is scored between 0–2, “0” being minimum and “2” maximum, with a minimum total score of “0” and maximum total score of “10” for all elements added together. In this system, weight loss is graded as “0” if <10% of original weight, “1” if between 10–25% and “2” if >25%. Posture, activity, fur texture and skin integrity are graded “0” if normal. Posture, activity and fur texture were graded as “1” or “2” depending on the level of hunching, decrease in activity or severity of skin ruffling, respectively. Skin integrity is graded “1” if scaling is seen on the paws or tail and “2” if the skin shows denuded areas. In parallel experiments, BMT recipient mice were sacrificed 6 days after BMT and subjected to analysis of cell composition by flow cytometry. GVHD-associated inflammation was graded by histopathology, as described below in detail.

### 4.4. Histopathology

On day 6 post-BMT, colons from BMT mice were fixed in 4% neutral buffered formalin and processed. Six μm sections were cut and stained with hematoxylin and eosin. Tissues were then analyzed for GVHD-related inflammation, and the severity of inflammation was scored in blinded fashion. GVHD-related colitis was scored as previously described [52]: as none (score of 0), mild (1), moderate (2), severe without ulcer (3) and severe with ulcer (4). Crypt apoptosis was graded as none (score of 0), fewer than 2 crypts per 10 containing an apoptotic body (1), 2–5 crypts per 10 containing an apoptotic body (2), the majority (>5) of crypts containing an apoptotic body (3) and the majority of crypts containing more than one apoptotic body (4). Scores ranged from 0 to 8.

### 4.5. Induction, Assessment of GVT and Quantification of Tumor Load

Luciferase-expressing A20 lymphoma (A20-luc) cells syngeneic with recipients (H2d) were used for these experiments. Each recipient mouse received 3 × 10^5^ A20-luc tumor cells i.v. within 24 h after BMT. Tumor load was assessed regularly in BMT recipient mice using an Ami 1000 Advanced Molecular Imager (Spectral Instruments, Tucson, AZ, USA) live animal imaging system. Five minutes before bioluminescent imaging, mice were placed in an oxygenated isoflurane chamber and administered d-luciferin (Promega, Madison, WI, USA) i.p. BMT recipient animals were imaged for 5 min, and tumor load was quantitated using Living Image software v2.50 (Caliper Life Sciences, Hopkinton, MA, USA).

### 4.6. Flow Cytometry

BALB/c recipient mice were sacrificed 6 days after BMT, and their spleens and mesenteric lymph nodes (MLNs) were isolated for analysis of cell composition. Spleens and MLN cells were also isolated from 5–8-week-old C57BL/6 and STAT6VT mice. For surface staining, 5 × 10^6^ cells were suspended in PBS with 2% FCS, and Fc receptors were blocked with 2.4G2 monoclonal antibody (mAb) (Clone: 93, BioLegend, San Diego, CA, USA). The following antibodies were used for surface staining: anti-CD4 FITC (Clone: GK1.5; Thermofisher, Waltham, MA, USA), anti-CD4 BV421 (Clone: RM4-5; BD Biosicences, Franklin Lakes, NJ, USA), anti-CD3 PE-Cy7 (Clone: 145-2C11; Thermofisher) and anti-H2b BV421 (clone AF6-88.5; BD Biosciences, San Jose, CA, USA). Intracellular staining for Foxp3 was performed using anti-Foxp3 APC (Clone: FJK-16S; Thermofisher) in accordance with the manufacturer’s instructions.

### 4.7. Cell Purification for In Vitro Cultures

CD3+ T cells were purified from spleens and MLNs of C57BL/6 or STAT6VT mice using a CD3 T cell isolation kit (Miltenyi Biotech); this resulted in >95% enrichment for CD3 T cells. Cells were stimulated with plate-bound anti-CD3 (clone: 145-2C11; eBioscience, San Diego, CA, USA) and soluble anti-CD28 (Clone: 37.51; eBioscience) (each at 1 μg/mL). IFNγ, TNFα, IL4 and IL10 were quantitated in supernatants of 48 h cultures, using antibody pairs from Thermofisher and according to the manufacturer’s instructions [6,10].

### 4.8. RNA Isolation and Bulk RNA Sequencing

RNA extraction was performed manually from sorted CD3+ T cells using the RNeasy Plus Mini Kit (QIAGEN, Germantown, MD, USA). RNA concentration was measured using NanoDrop 2000c (Thermo Scientific, Waltham, MA, USA) and RNA quality was assessed via the RNA 6000 NanoKit for the Bioanalyzer 2100 (Agilent Technologies, Santa Clara, CA, USA). High-quality RNA samples (RNA integrity number > 8) were used for the gene expression analysis by RNA-seq. Sequencing libraries were prepared from 125 ng of DNase I-treated total RNA, using the Illumina stranded mRNA library preparation kit (Cat. #20040534, Illumina, Inc., San Diego, CA, USA). The molar concentrations of the resulting indexed libraries were measured using the 2100 Agilent Bioanalyzer (Agilent Technologies, Santa Clara, CA, USA) and combined equally into a pool for sequencing. The concentration of the library pool was measured using the Illumina Library Quantification Kit (KAPA Biosystems, Wilmington, MA, USA) and sequenced on the Illumina NovaSeq 6000 genome sequencer using 100 bp paired-end SBS chemistry.

### 4.9. Bioinformatic Analysis

Publicly available STAT6 ChIP-Seq bedgraph files were downloaded from GSE22104 [23] and converted to BigWig files for viewing on the Integrated Genome Viewer (IGV) Browser using the murine annotation genome MM9. For analysis of bulk RNA-seq data, barcoded samples were pooled and sequenced using an Illumina NovaSeq 6000 in the Iowa Institute of Human Genetics (IIHG) Genomics Core Facility. Paired-end reads were demultiplexed and converted from the native Illumina BCL format to fastq format using an in-house Python wrapper to Illumina’s “bcl2fastq” conversion utility. FASTQ data were processed with nf-core/rnaseq (v3.12), a best-practices pipeline available at the open-source “nf-core” project (Version: Revision 3bec2331ca [3.12.0]) (https://nf-co.re/ (accessed on 6 May 2024), Nextflow version 22.10.1). Reads from the samples were aligned against the Ensembl reference “GRCm38” using the STAR aligner. Concurrently, reads were also pseudo-aligned to the transcriptome using the Salmon aligner [53] (Salmon performs its own internal quantitation, yielding estimated counts and values in length-normalized TPM (transcripts per million)). Transcript-level abundances were converted to gene-level counts. Quality control was performed with qualimap and multiQC, a computational tool that detects common QC problems [54,55,56,57]. Gene-level counts were used for differential gene expression analysis with DESeq2 [58]. Bioconductor package “PCAExplorer” (Software Version: pcaExplorer_2.28.0) was used for exploratory analysis [59]. The DE gene lists were analyzed using Advaita Bio’s iPathwayGuide; Software Version: 17.1 (https://www.advaitabio.com/ipathwayguide (accessed on 15 October 2024)). This software analysis tool implements the “Impact Analysis” approach that takes into consideration the direction and type of all signals on a pathway [60,61,62,63] The raw FASTQ files and associated metadata have been made available for download at GEO accession XYZ.

### 4.10. Statistics

Differences in survival between groups were determined by Kaplan–Meier’s log-rank test. Differences in cell composition, disease score, weight change, cytokine content and histopathological GVHD scores between infected and uninfected groups were determined using the GraphPad Prism software (Version 10.2.3) and significance was determined using *t*-test. Difference in tumor development between the three groups was calculated using Fisher’s exact test with Bonferroni correction and using an adjusted *p*-value of 0.05/3 = 0.017.

## 5. Conclusions

Our results attest to a novel, antitumor immunity generated by constitutive STAT6 activity in donor T lymphocytes in BMT mice. STAT6 drives the expression of anti-tumor effector proteins, such as IFNγ or FasL, and directly stimulates the expression of other anti-tumor effectors, such as granzyme A or granzyme B. Last, STAT6 activates donor T cell Th2 pathway and regulatory cytokine production and thereby has an impact on generation/expansion of GVHD-regulating donor T lymphocytes. The major impact of constitutive STAT6 activity on donor T cells after BMT is the preservation of a robust GVT effect without causing GVHD, which requires further attention to better understand how T cells mechanistically separate the GVT effect from GVHD and characterize its translational potential.

## Data Availability

The data presented in this study are available on request from the corresponding author.

## Supplementary Materials

The following supporting information can be downloaded at: https://www.mdpi.com/article/10.3390/ijms26010280/s1.
