# Supplementary material for: The Impact of Cell-Intrinsic STAT6 Protein on Donor T Cell-Mediated Graft-Versus-Tumor Effect
Source: Int J Mol Sci. 2024 Dec 31;26(1):280. doi: 10.3390/ijms26010280 (PMC11719522; doi:10.3390/ijms26010280)
Supplement: Supplementary file 1 [file ijms-26-00280-s001.zip › ijms-3305334-supplementary.pdf]

Supplementary Data to Figure 1 (Guan et al.)

Supplementary data to Figure 1C. Difference in disease scores between two groups (BM: Recipients of TCD-BM cells only; VT+: recipients of VT+ donor T cells; VT-: recipients of VT- donor T cells). Difference in significance (p value) as indicated on each time point between groups; NS: nonsignificant.

Days	7	14	21	28	35	42	49	56	63	70
BM only vs BMT VT+	NS	<0.05	<0.05	NS	<0.05	<0.05	<0.05	<0.05	NS	<0.05
BM only vs BMT VT-	<0.0001	<0.0001	<0.0001	<0.0001	<0.0001	<0.0001	<0.0001			
BMT VT+ vs BMT VT-	<0.0001	<0.0001	<0.0001	<0.0001	<0.0001	<0.0001	<0.0001			

Supplementary data to Figure 1D. Difference in weight between two groups (BM: Recipients of TCD-BM cells only; VT+: recipients of VT+ donor T cells; VT-: recipients of VT- donor T cells). Difference in significance (p value) as indicated on each time point between groups; NS: nonsignificant.

Days	7	14	21	28	35	42	49	56	63	70
BM only vs BMT VT+	<0.05	NS	NS	NS	NS	NS	NS	NS	NS	NS
BM only vs BMT VT-	<0.05	<0.0001	<0.0001	<0.0001	<0.0001	<0.0001	<0.0001			
BMT VT+ vs BMT VT-	<0.001	<0.0001	<0.0001	<0.0001	<0.0001	<0.0001	<0.0001			
